# Pressure-Dependent Elevation of Vasoactive Intestinal Peptide Level in Chicken Choroid

**DOI:** 10.3390/biology12040495

**Published:** 2023-03-24

**Authors:** Evgeny Privalov, Matthias Zenkel, Ursula Schloetzer-Schrehardt, Stefanie Kuerten, Antonio Bergua, Bettina Hohberger

**Affiliations:** 1 Department of Ophthalmology, Universität of Erlangen-Nürnberg, Friedrich-Alexander-University-Erlangen-Nürnberg (FAU), 91054 Erlangen, Germany; 2 Institute of Neuroanatomy, Medical Faculty, University of Bonn, 53115 Bonn, Germany

**Keywords:** tissue pressurization, vasoactive intestinal peptide, choroid, intraocular pressure, glaucoma, ICN

## Abstract

**Simple Summary:**

Autonomic ocular control is mediated by sympathetic, parasympathetic, and primary trigeminal afferent nerve fibers. Intrinsic choroidal neurons (ICN) contribute to this complex neuronal network. Vasoactive intestinal peptide (VIP), a major transmitter of ICN, mediates choroidal vasodilation and, thus, potentially choroidal thickness and intraocular pressure (IOP). Therefore, it was the aim of the present study to investigate the choroidal VIP level (VIP_chor_) in the presence of an increased atmospheric pressure in a chicken model. Chicken choroidal whole mounts were exposed to ambient pressure (*n* = 20) and 40 mmHg (*n* = 20) in a PC-controlled, open chamber system for 24 and 72 h, respectively. VIP_chor_ was significantly increased after exposure to 40 mmHg compared to exposure to ambient pressure. This increase of the VIP_chor_ level, representing the intracellular choroidal VIP level, might argue for a retention of VIP within the neurons, consequently decreasing vasodilatation and choroid thickness.

**Abstract:**

Purpose: Autonomic control is important in maintaining ocular integrity. As recent data suggested that intrinsic choroidal neurons (ICN), an intrinsic choroidal autonomic control, may regulate choroidal thickening via release of the vasodilative vasoactive intestinal peptide (VIP), it was the aim of the study to investigate the level of choroidal VIP (VIP_chor_) in the presence of an increased atmospheric pressure in a chicken model. Methods: Chicken choroidal whole mounts were exposed to ambient pressure (*n* = 20) and 40 mm Hg (*n* = 20) in a PC-controlled, open chamber system for 24 and 72 h, respectively. The VIP concentration was analyzed by ELISA, and the total protein concentration was measured by the BCA assay. Statistical analysis was done using an unpaired two-tailed *t*-test. Results: The pressurization systems enabled choroidal whole mount pressurization (40 mm Hg) with humidifying, pressure, temperature, and gas exchange. Overall, the VIP_chor_ level concentration was significantly increased at 40 mmHg compared to the ambient pressure (30.09 ± 7.18 pg vs. 20.69 ± 3.24 pg; *p* < 0.0001). Subgroup analysis yielded a significantly increased VIP_chor_ level at 40 mmHg compared to the ambient pressure after 24 h (28.42 ± 6.03 pg vs. 20.76 ± 4.06 pg; *p* = 0.005) and 72 h (31.77 ± 7.82 pg vs. 20.61 ± 2.12 pg; *p* = 0.002), respectively. The VIP_chor_ elevation at 40 mm Hg ranged between 1.37- (24 h) and 1.54-fold (72 h) compared to the ambient pressure. No difference was observed between the VIP_chor_ level at 24 h and 72 h (*p* > 0.05). Conclusions: The increase of the total choroidal VIP level, representing the intracellular VIP content, in the presence of an increased ambient pressure argues for a retention of VIP within the neurons, decreasing both vasodilatation and, consequently, choroid thickness. This finding might be a passive or even active function of ICN in the regulation of choroidal thickness, ocular integrity and IOP.

## 1. Introduction

Autonomic control of the eye is mediated by a complex neuronal meshwork of sympathetic [1] and parasympathetic fibers [2,3,4] and primary afferent nerve fibers of the trigeminal nerve [5]. This neuronal network regulates accommodation, pupil size and ocular blood flow [6,7,8,9]. Furthermore, intraocular pressure (IOP) and aqueous humor (AH) production are controlled by fine autonomic innervation [9,10]. Intrinsic choroidal neurons (ICN) are assumed to be a fourth important autonomic component of ocular neuronal regulation [11]. 

ICN were described in 1859 for the first time [12]. These neurons were localized within the choroid, mainly co-expressing the transmitters, vasoactive intestinal polypeptide (VIP) and nitric oxide (NO) [13,14,15]. A topographic analysis yielded a temporal and suprachoroidal accumulation [5,13,16,17,18]. ICN are assumed to contribute to the complex neuronal autonomic network within the choroid analog to the myenteric plexus (Auerbach Plexus) [19]. Interestingly, only humans and species with high visual acuity (i.e., birds) show large amounts of ICN [20,21]. As classical animal models (e.g., rat and mouse) lack or even show low numbers of ICN [14,22,23], the avian model seems to be predisposed toward analysis of ICN [5,21,24]. The function of ICN has been elusive until now. Close neuronal contacts of ICN were observed for blood vessels, non-vascular smooth muscle cells (NVSMC) and choroidal melanocytes [20,25,26,27]. It is supposed that ICN are involved in ‘choroidal accommodation’ and the regulation of choroidal blood flow [28,29,30]. Considering this topographic aspect and their content of transmitters, ICN might regulate vessel diameters (vasodilation) and the consecutive choroidal thickness. Recent data suggest that the regulation of choroidal thickness might attribute to IOP control [16]. A circadian analysis of VIP concentration yielded an increased level in the evening compared to the morning [16]. This VIP release over the night is parallel with the circadian changes of the choroid (i.e., increased during the night) [31] and antithetical changes of IOP (i.e., lower at night) [32]. It can be assumed that this nocturnal VIP release contributes to the regulation of ocular integrity by choroidal thickening as compensation of a lower IOP. Circadian changes of IOP were shown in several studies throughout different species, including humans [33,34,35], birds [32], smaller mammals [36] and rodents [37]. 

IOP regulation is complex and still under investigation. The level of IOP is the steady state of the production in the nonpigmented ciliary body and outflow of the aqueous humor (AH, trabecular meshwork and suprachoroidal) [38]. As (I) circadian changes of the IOP, choroidal thickness and level of VIP were known, and (II) a link between the level of IOP and VIP was supposed, it was the aim of the present study to investigate the level of choroidal VIP (VIP_chor_) dependent on different levels of ambient pressure in an in vitro avian model.

## 2. Materials and Methods

### 2.1. Tissue

Forty eyes of 20 chickens (*Gallus domesticus*, *type Ross*, aged 6–8 weeks) were enucleated after slaughtering, which was performed for commercial purposes at a local farm. 

All actions concerning the animals were performed according to the European and national legislation for animal welfare [39,40]. The animals were slaughtered between 8 a.m. and 10 a.m. to avoid circadian fluctuations in the VIP concentration within the obtained tissues. Choroidal whole mounts were prepared in DMEM/Ham’s F12 medium.

### 2.2. Preparation of Choroidal Whole Mounts

Choroidal whole mounts were prepared as described earlier [16]. Briefly, the eyeballs were opened along the ora serrata, and the retinal pigment epithelium, retina and vitreous body were removed by blunt tweezers. If any retinal pigment epithelium remained, this tissue was removed carefully by cotton swabs. Choroidal whole mounts were prepared after separation of the choroids from the sclera and pecten oculi using tweezers and scissors to cut the connecting structures. The tissues were rinsed in phosphate-buffered saline (PBS; PAN Biotech, Aiden Bach, Germany). All choroids were transferred to a 6-well plate containing 8 mL of culture medium (DMEM/HAM’s F12 with 8 g/L glucose and 1× N2 supplement; Thermo Fisher Scientific, Schwerte, Germany) and incubated at 37 °C. 

### 2.3. Pressurization of Choroidal Whole Mounts

The experiments were done subdividing the choroidal whole mounts into two groups: (1) exposed to ambient pressure (*n* = 20, pressure group) and (2) exposed to a pressure of twice the upper range of a regulated human IOP (i.e., 40 mm Hg, *n* = 20, control). Considering a time-dependent, yet not a circadian, effect, experiments with choroidal whole mounts were performed for 24 h (*n* = 10) and 72 h (*n* = 10), respectively. Keeping a constant temperature of 37 °C, the experiments were performed within an incubator. After 24 h, the culture medium was replaced. Considering inter-individual differences in tissue compositions, the choroidal whole mounts of the left eye were assigned to the pressure group and that of the right eye to the control group. 

### 2.4. Enzyme-Linked Immunosorbent Assay

After the incubation, the choroidal whole mounts were homogenized by the Precellys 24 homogenizer and lysing kit (Bertin; Frankfurt, Germany) in PBS with 3 cycles of 2 × 30 s at 5000 rpm. The choroidal homogenates were centrifuged at 14,000 rpm (21,913× *g*) for 10 min (4 °C), and the supernatant was frozen immediately at −80 °C. VIP concentrations in the supernatant were analyzed using an enzyme-linked immunosorbent assay, namely the Chicken VIP Peptide ELISA Kit (Chicken VIP Peptides ELISA Kit, WUHAN EIAAB SCIENCE CO., LTD, Wuhan, China) according to the manufacturer’s protocol. The total protein concentration was measured by the Micro BCA Protein Assay (Thermo Fisher Scientific, Schwerte, Germany). The VIP and total protein levels were measured using the MULTISCAN SPECTRUM and Skanlt software V 2.2 (both by Thermo Fisher Scientific, Schwerte, Germany).

### 2.5. Statistical Analysis

Normal Gaussian distribution was tested using the Kolmogorov–Smirnov test. Group comparisons were performed using an unpaired two-tailed *t*-test. A *p*-value of <0.05 was considered statistically significant. Choroidal VIP concentrations were presented as the mean ± standard deviation. Statistical power analysis was performed to determine the sample size needed for significant data and yielded a minimum of 6 specimen (alpha: 0.05, power: 90%).

## 3. Results

### 3.1. Pressurization System

**Figure 1:**
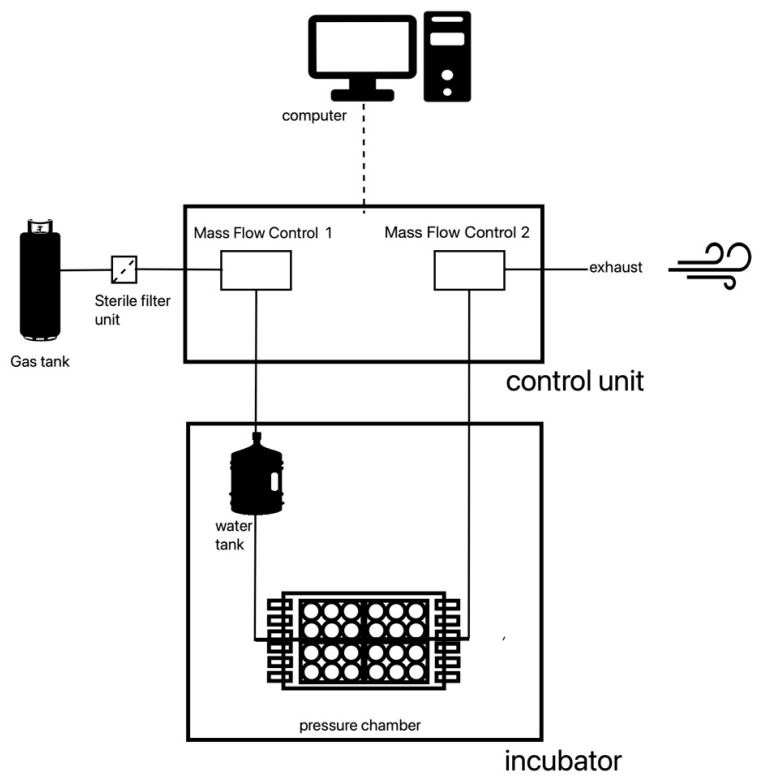
Pressurization system—schematic illustration.

**Figure 2:**
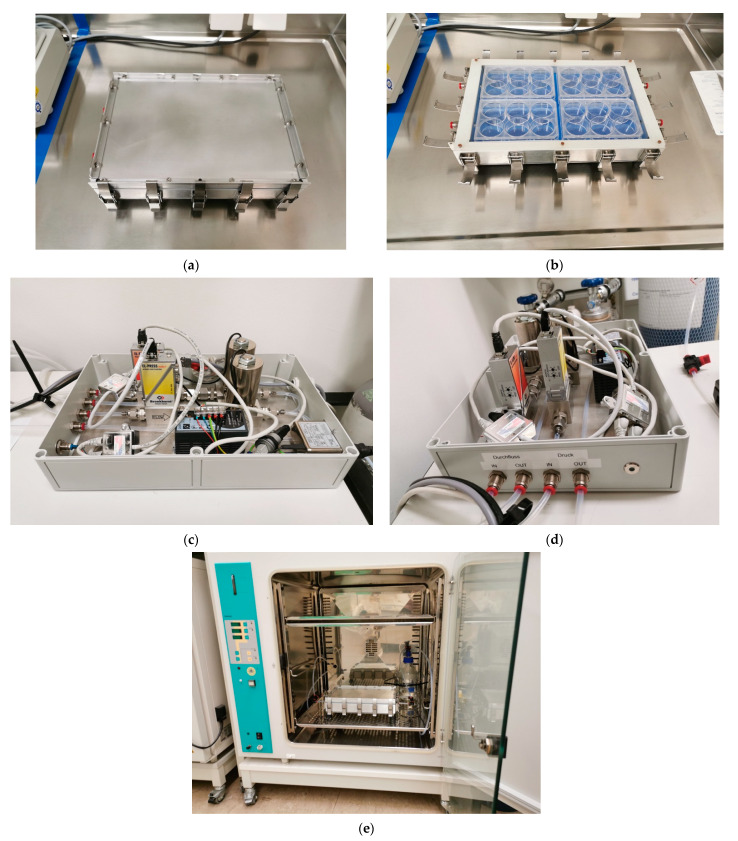
Pressurization system. (**a**) Pressurization chamber with lid; clasps closed. (**b**) Pressurization chamber without lid; clasps open. (**c**) Pressurization chamber control unit. (**d**) Pressurization chamber control unit; German translations: Druck = pressure, Durchfluss = flow. (**e**) Pressurization chamber inside of an incubator.

The construction of the pressurization system was done within the scope of contract work at the Department of Process Machinery and Plant Engineering Friedrich-Alexander-University-Erlangen-Nürnberg (FAU). The pressurization system consists of four parts (Figure 1 and Figure 2e). The chamber itself houses four 6-well-plates; a gas container (comprising a gas mixture of 74% nitrogen, 21% oxygen and 5% carbon dioxide); the control unit that regulates the flow of gas and a computer. The specially designed software operates the system and documents the pressure and the temperature every minute. 

The gas is released from its container through a two-step pressure reducer. Before entering the chamber, the gas passes through a sterile filter, the first MFC vent (mass flow control) and a water tank. Then, the humidified and warmed gas enters the chamber. Immediately after the chamber, the gas passes a second MFC vent. The outflow of the second MFC vent is controlled by the computer. Through this outflow control, the pressure of 40 mm Hg is maintained consistently over an extended period. To ensure that the fluctuations in pressure are at a bare minimum, a computer program, which supervised the experiment, recorded the pressure in the system every minute. If a deviation of 1 mm Hg over a period longer than one minute was recorded, this section of the experiment was repeated. The pressure chamber is placed in an incubator to maintain a constant temperature of 37 °C.

### 3.2. VIP_chor_ Concentration

The total VIP_chor_ concentration was significantly increased at 40 mm Hg compared to the ambient pressure (*p* < 0.0001; Table 1). We observed the overall VIP_chor_ to be increased 1.45-fold at 40 mmHg compared to the ambient pressure (Table 1).

The subgroup analysis yielded a significantly increased mean VIP_chor_ concentration after 24 h at 40 mmHg (28.42 ± 6.03 pg VIP/µg total protein) compared to the ambient pressure (20.76 ± 4.06 pg VIP/µg total protein, *p* < 0.005). In addition, the mean VIP_chor_ concentration was significantly increased after 72 h of pressure incubation at 40 mmHg (31.77 ± 7.82 pg VIP/µg total protein) compared to the ambient pressure (20.61 ± 2.12 pg VIP/ug total protein; *p* = 0.002). The VIP increase was 1.37-fold (after 24 h) and 1.54-fold (after 72 h) at 40 mm Hg compared to the ambient pressure (Table 1). The VIP_chor_ concentration (72 h) was not significantly different compared to the VIP_chor_ level (24 h) in the presence of 40 mm Hg (*p* > 0.05).

## 4. Discussion

Intraocular pressure is known to fluctuate over a 24-h period [32,34,41]. Although fluctuations of a few mm Hg are considered normal [32], the maintenance of a constant IOP is paramount to sustain ocular homeostasis, function and efficiency [38,42]. These circadian fluctuations of IOP have been described in humans [33,34] and throughout different animals [36,37]. In young chickens, IOP tends to decrease during the night and reach its peak during the day [32,43]. Experimental studies showed that choroidal thickness [31,44] and the release of VIP by ICN [16] follow the circadian rhythm in a chicken model. 

Considering VIP as a potent vasodilator, consecutively increasing the choroidal blood flow [45,46,47], the aim of this study was to investigate the level of choroidal VIP in the context of different ambient pressures in an in vitro chicken model. Exposing choroidal whole mounts to a pressure of 40 mm Hg, we observed a significantly increased overall mean VIP_chor_ concentration compared to the ambient pressure (1.37-fold). This increase of the mean VIP_chor_ concentration lasted over the whole experimental observation period of 72 h compared to the ambient pressure (1.54-fold). 

A multitude of laboratory devices have been developed for the treatment of cells and different human and animal tissues with increased ambient pressure in order to investigate their response to hypertensive conditions [48]. Commercially available systems providing pressure, temperature and gas exchange control are often not in the budget for small laboratories. A simple and inexpensive way to exert pressure stress is a pressurized chamber designed as a closed system [49]. However, closed systems do not provide gas exchange, leading to hypoxic conditions and pH shifts in culture mediums [50]. The design of our self-constructed pressurization system provides pressure control and a constant temperature, humidification, and gas exchange. Therefore, this device may be an affordable alternative to high-priced commercial systems.

Chicken models have already been used in a variety of different ophthalmological research topics, such as corneas disease, retinopathies, ophthalmological tumors, or glaucoma [51,52]. Especially, light-induced glaucoma (LIG) has been studied in chicken models [53,54,55,56]. Of interest, the use of avian models in the research of ICN was first established by Bergua et al. [5] by introducing the duck *Carina moschata.* This experimental study provided the first evidence of similar numbers of ICN in avian choroids compared to humans. 

Subsequent studies yielded similar amounts of ICN in different avian models, yet not in rats, mice, or rabbits [5,11,14,20]. Several homologies between human and chicken ICN predisposed chicken as the animal model in the research of ICN: the basic anatomic characteristics (i.e., shape and choroidal localization); neuronal synaptic interactions (sympathetic, parasympathetic, trigeminal neurons and inter-neuronal ICN interactions); major transmitters (VIP and NO) [13,14] and targets (blood vessels and NVSMC) [11,20,24,25,57,58]. 

First described by Müller in 1959 [12], ICN contribute to the complex autonomic ocular control system. Parasympathetic input is mediated by the ciliary [2,5] and the pterygopalatine ganglia (PPG) [3,58], while the superior ciliary ganglion (SCG) contributes to sympathetic choroidal innervation [1,4]. In addition, the primary afferent nerve fibers of the trigeminal ganglion provide neuronal input to this autonomic regulation [5]. Similar to other organs (e.g., the gut) [59], the choroid seems to have its own intrinsic neurons [16,30,60], yet, their specific function is still elusive. As their major neurotransmitters (VIP and NO) are known to be vasoactive agents [15,45,46], a regulation of the choroidal blood flow [20] and, thus, choroidal thickness [32,61] and, potentially, the IOP can be assumed [6,16,20,47]. 

VIP is of special interest, as it was observed in the PPG and the ICN, providing a special form of intrinsic and extrinsic regulation [28]. VIP, first described in 1970 [62], was characterized in the porcine intestine as a 28 amino acid residue polypeptide with systemic vasodilation properties. VIP is attributed to the PACAP (pituitary adenylate cyclase-activating peptide) family [63], with its gene completely analyzed in 1985 by Tsukada [64]. Showing strong structural similarities to glucagon and secretin, VIP is derived from a precursor protein, namely the Pre-Pro VIP [65]. The Pre-Pro VIP gene is found on chromosome 6 in humans (6q25.2), has a length of 9 kb and is divided by six introns [66]. These introns code for the known cleavage products within the Pre-Pro-VIP, such as the 5’untranslated region and PHM (exon 4). The VIP itself is encoded on exon 5. Due to the presence of an intron within the 3’untranslated region, it is also assumed that the introns of the gene divide the precursor peptide into functional zones [64,67]. PHM is considered to be the human counterpart to PHI-27 [62]. PHI-27, being expressed in the porcine intestines [68], acts as an inhibitor of the binding of VIP to its receptors [69]. VIP can activate three G-protein-coupled receptors (GPCR; VPAC1, VPAC2 and PAC1). VPAC1 and VPAC2 induce the cAMP-PKA pathway, whereas PAC1 mediates their functional activity via PKA and PLC [67,70]. 

Overall, VIP is known to be a potent neurotransmitter further involved in the regulation of circadian rhythms [71], hormones [72,73], the intestines [74,75] and cancer [76,77,78]. As these GPCRs were observed in different immune cells (e.g., macrophages, CD4+ T cells and CD8+ T cells lymphocytes) [79,80,81], an involvement in the immune response can be assumed. 

Focusing on the function of VIP within the choroid, smooth muscle relaxation and vasodilation are characteristics predisposing a regulatory function within the choroidal blood flow [13,14,15]. VIP-positive neurons were observed to form a perivascular plexus around arteries in ducks [5]. The presence of a single base membrane and a full glia coating provide morphological findings for an additional mechanosensory function [16,25]. Furthermore, ICN were observed in regions underlying retinal areas with high visual acuity (subfoveal) [14,21]. It is assumed that ICN contribute to choroidal accommodation by their quick intrinsic plexus with a dual function via a passive (mechanosensory) and an active (vasodilative) component [20,82,83]. Regulatory interactions between nerve cells and the vasculature are also known in the central nervous system, as microglia were shown to regulate the cerebral blood flow (CBF) through PANX1-P2Y12 coupling and contribute to the “vascular reactivity” of the CBF [84].

Considering these different aspects and a correlation between the blood flow and IOP [6], we assume there is a link between the VIP, choroidal vasodilation, blood flow and IOP. The choroid is a dense vascular network responsible for about 85% of the ocular vascular supply [85,86]. 

Clinical studies offer that choroidal thickening might compensate (passive) or even influence (active) IOP [86,87,88,89,90]; thus, a link between IOP (i.e., ambient pressure in in vitro experiments) and choroidal thickness (i.e., choroidal VIP as correlated for vasodilative function) can be assumed. Patients with an increased IOP, namely ocular hypertension, showed a choroidal thinning [91,92]. Decreasing the IOP by a surgical method (trabeculectomy) resulted in an increase of subfoveal and peripapillary choroidal thickness in patients with increased IOP and neurodegeneration of the optic nerve head (glaucoma) [90,93,94,95,96]. Interestingly, subfoveal choroidal thickness was observed to decrease significantly in the presence of an acute IOP increase (time range 2 h) in healthy humans, probably mediated by a fast molecular mechanism [88]. 

## 5. Limitations of the Study

The present study is not without limitations. The present experimental set-up was performed as an in vitro experiment in a chicken model. Thus, we used atmospheric pressure (as correlated for IOP) and an animal model for the analysis of VIP in ICN research. This set-up does not claim to represent the complex dynamic physiological conditions (e.g., blood flow and hormones) within an in vivo chicken or even human eye. Further studies are necessary to investigate the clinical implications of VIP in regulating IOP and choroidal thickness.

## 6. Conclusions

In the present study, we observed an increase in the total choroidal VIP level, representing the intracellular VIP content, in the presence of an increased ambient pressure. Thus, we assume that VIP is retained within the neurons, decreasing both vasodilation and, consequently, the choroid thickness. This finding might be a passive or even active function of ICN in the regulation of choroidal thickness, ocular integrity and IOP. 

## Figures and Tables

**Table 1:** VIP_chor_ concentration after incubation at the ambient pressure (control) and 40 mm Hg for 24 h and 72 h: mean VIP_chor_ level (pg/ug total protein) and standard deviation; *n*—sample size; Chicken VIP Peptides ELISA Kit, WUHAN EIAAB SCIENCE CO., LTD, Wuhan, China, was used.

Group (*n*)	Time (Hour)	Pressure (mmHg)	VIP (Mean ± SD)	Fold Change	*p*-Value
Control (20)	compounded	ambient	20.69 ± 3.24		
Pressure (20)		40	30.09 ± 7.18	1.45	<0.0001
Control (10)	24	ambient	20.76 ± 4.06		
Pressure (10)	24	40	28.42 ± 6.03	1.37	0.005
Control (10)	72	ambient	20.61 ± 2.12		
Pressure (10)	72	40	31.77 ± 7.82	1.54	0.002

## Data Availability

Not applicable.
